# Editor’s introduction to this issue (G&I 20:3, 2022)

**DOI:** 10.5808/gi.20.3.e1

**Published:** 2022-09-30

**Authors:** Taesung Park

**Affiliations:** Department of Statistics, Seoul National University, Seoul 08826, Korea

This issue contains one 10 original articles and one application note.

Among the 10 original articles, there were six clinical studies, one vaccine-related study, one on drug design, one about multilevel analysis, and one about a Java pipeline for the mining and analysis of microsatellites in human coronavirus genomes. First, Dr. Asmy (Bharathiar University, India) considered the microvascular complications of diabetes, such as diabetic retinopathy, neuropathy, and nephropathy. The authors identified the weighted co-expressed and differentially expressed genes (DEGs), major pathways, and their miRNAs, transcription factors (TFs), and drugs interacting in all three conditions. The authors successfully demonstrated the integrative identification of biomarker genes, miRNAs, TFs, and therapeutic drugs in key signaling pathways, helping to better understand the processes of all three secondary microvascular problems and aiding in disease detection and management. Specifically, the authors first identified five overlapping genes (*AKT1, NFKB1, MAPK3, PDPK1*, and *TNF*) from the DEGs and co-expressed genes as key genes. They then constructed the miRNA-gene and TF-gene regulatory networks of the five genes in the nine major pathways. Then, they showed that a major miRNA (hsa-mir-34a-5p) interacted with all five genes. Next, they showed that RELA, FOXO3, PDX1, and SREBF1 were the TFs interacting with the major five genes of interest. Finally, through drug-gene interaction network analysis, the authors suggested five potential drugs to treat the genes of interest.

The work led by Dr. Shojaei (Hamadan University of Medical Sciences, Iran) was about oral squamous cell carcinoma (OSCC), which is the most prevalent head and neck malignancy. The authors identified potential markers, including miRNAs and genes, significantly involved in the etiology of early-stage OSCC. The authors identified a total of 23 differentially expressed miRNAs in patients with primary OSCC compared to the healthy controls, which targeted genes including *CALM1, CYCS, THBS1, MYC, GATA6, SPRED3, PIK3R3, GIGYF1*, and *BCL2L11*. The present study revealed a possible mechanism mediating primary OSCC and may be useful for predicting the prognosis of patients with early-stage OSCC.

Dr. Zavarzadeh (University of Tabriz, Iran) presented a clinical, laboratory, and genetic study of a pathogenic variant of the *CYP1B1* gene using whole-exome sequencing data from a rare case of primary congenital glaucoma. Dr. Rha (Yonsei University College of Medicine, Korea) and colleagues evaluated the frequencies of *UGT1A* polymorphisms and their relationship with clinicopathologic parameters in 382 Korean gastric cancer patients. Polymorphisms of UGT1A1*6, UGT1A1*27, UGT1A1*28, UGT1A1*60, UGT1A7*2, UGT1A7*3, and UGT1A9*22 were genotyped. While many clinically important findings were made, the most clinically important finding was about UGT1A9*22. The genotype of UGT1A9*22 polymorphisms was shown to identify high-risk patients, among gastric cancer patients receiving irinotecan-containing chemotherapy and suffering severe toxicity. Thus, when treating high-risk patients with UGT1A9*22 polymorphisms, clinicians might closely monitor them for severe manifestations of toxicity, such as intense diarrhea or neutropenia.

Next, Dr. Salehi (Isfahan University of Medical Sciences, Iran) identified two novel mutations in *ALDH18A1* (located on 10q24.1) and *SPG11* (located on 15q21.1) by whole-exome sequencing in hereditary spastic paraplegia patients in Iran. Dr. Raza (University of Chittagong, Bangladesh) presented a systemic study on the vulnerability and fatality of prostate cancer patients towards coronavirus disease 2019 (COVID-19) through an analysis of *TMPRSS2, CXCL10*, and their co-expressed genes. Dr. Dey (University of Dhaka, Bangladesh) and collaborators performed an analysis of gene expression profiles to study malaria vaccine dose efficacy and immune response modulation. They used gene expression profiles of pre- and post-vaccination patients after various doses of the RTS,S vaccine based on samples collected from the Gene Expression Omnibus datasets.

Dr. Islam’s group (Jashore University of Science and Technology, Bangladesh) focused on nervous necrosis virus (NNV), evaluating the inhibitory potential of 70 compounds of *Azadirachta indica* (Neem plant), which has been reported to show potential antiviral activity against NNV. The binding affinity of 70 compounds was calculated against the grouper heat shock cognate protein 70 with docking and molecular dynamics simulation approaches. Neem plant compounds may act as significant inhibitors of viral entry into the host cell.

Next, joint work with Dr. Park (Indiana University School of Medicine, USA) and Dr. Chung (Kyonggi University, Korea) was about the missing data in multilevel analyses. It is well known that multilevel analysis is an appropriate and powerful tool for analyzing hierarchically structured data widely applied from public health to genomic data. Quite often, there may be a loss of information on multiple nesting levels in a multilevel analysis. Park and Chung considered a multilevel linear mixed effect model (LMM) with single imputation with all data hierarchy levels in the presence of missing top or intermediate-level clusters. They evaluated and compared the performance of a multilevel LMM with single imputation with other models ignoring the data hierarchy or missing intermediate-level clusters. Through simulation studies, they demonstrated that an LMM with single imputation estimates fixed the coefficients and variance components of a multilevel model more accurately than other models ignoring data hierarchy or missing clusters in terms of mean squared error and coverage probability.

The final original article is about a pipeline. Dr. Umang (Shri Venkateshwara University, India) proposed using an in-house built Java pipeline to identify, analyze, design primers, and find related statistics of perfect and compound microsatellites in seven complete genome sequences of coronaviruses, including COVID-19, the host of which is *Homo sapiens*.

Finally, there is one application note regarding NBLAST, which is a graphical user interface-based two-way BLAST software. BLAST has been one of the most widely used bioinformatics programs. However, for large sequence data, the web-based BLAST program often suffers from a problem of upload size limitation. To overcome this issue, Dr. Choi (Kangwon National University, Korea) developed NBLAST, which allows the use of input sequences either as “query” or “target” in the BLAST analysis. NBLAST is also equipped with a dot plot viewer.

